# Hyperprolactinemia with antipsychotics and the need for magnetic resonance imaging

**DOI:** 10.1192/j.eurpsy.2022.1038

**Published:** 2022-09-01

**Authors:** M.D.C. Molina Liétor, I. Cuevas Iñiguez

**Affiliations:** 1 Hospital Universitario Príncipe de Asturias, Psiquiatría, Alcalá de Henares, Spain; 2 Hospital Principe de Asturias, Psiquiatría, Alcala de Henares, Spain

**Keywords:** BIPOLAR, magnetic resonance imaging, Hyperprolactinemia, risperidone

## Abstract

**Introduction:**

Hyperprolactinemia is a frequent medical condition in daily clinical practice. In most laboratories, normal prolactin (PRL) concentrations are less than 25ng / ml in women and less than 20ng / ml in men. The causes of hyperprolactinemia can be physiological or secondary, among which a differential diagnosis must be made.

**Objectives:**

The causes of hyperprolactinemia are reviewed on the basis of a clinical case.

**Methods:**

Bibliographic review and presentation of a clinical case.

**Results:**

The case of a 17-year-old patient is presented, who comes to the Emergency Department due to a picture of agitation at home. Her relatives comment that two months ago, they began to notice her strange, very active and without the need to sleep. During the examination, the patient presents with verbiage, flight of ideas, and megalomanic thoughts. A manic episode was diagnosed and the patient was admitted to the psychiatric hospital. She was prescribed risperidone up to 4mg / day, carrying out prolactin determination after a few days. The baseline prolactin determination was 140 ng / ml and 130 ng/ml at twenty minutes. Due to the very high levels, the question arises as to whether the cause of hyperprolactinemia is due to treatment or hypothalamic damage. The MRI: “slight asymmetry in the pituitary gland, being discreetly more globular the adenohypophyseal LD, which could be in relation to underlying microadenoma”. As there were no previous data, the decision was made to withdraw risperidone with the introduction of aripiprazole and imaging tests periodically.

**Conclusions:**

Differential diagnosis of the cause of hyperprolactinemia is important.

**Disclosure:**

No significant relationships.

